# Case Report: Acute effect of benralizumab on asthma exacerbation without concomitant corticosteroid use

**DOI:** 10.12688/f1000research.24603.2

**Published:** 2020-07-23

**Authors:** Santi Nolasco, Raffaele Campisi, Rossella Intravaia, Morena Porto, Corrado Pelaia, Nunzio Crimi, Claudia Crimi

**Affiliations:** 1 Department of Clinical and Experimental Medicine, Section of Respiratory Diseases, University of Catania, Catania, Italy; 2 Respiratory Medicine Unit, A.O.U. “Policlinico-Vittorio Emanuele”, Catania, Italy; 3 Department of Medical and Surgical Sciences, University “Magna Graecia”, Catanzaro, Italy

**Keywords:** anti-IL-5 antibody, asthma control test, benralizumab, eosinophilic asthma, speed of onset, severe refractory asthma, asthma exacerbation

## Abstract

**Background**: Monoclonal antibodies are a relatively new therapeutic option for patients with severe refractory asthma, which can be used as an add-on to maintenance therapy, reducing the need for systemic corticosteroid usage, improving asthma symptom control and reducing exacerbations. We report a case of a patient with severe refractory eosinophilic asthma, reluctant to take systemic steroids, who was successfully treated with benralizumab alone during an acute asthma attack.

**Case presentation**: A 59-year-old Caucasian woman with a history of allergic asthma since childhood showed a progressive decline in lung function with difficult to control symptoms and an increased number of hospitalizations despite maximal maintenance treatment, and was diagnosed with severe refractory asthma. She was reluctant to take systemic corticosteroids during exacerbations due to severe urinary retention; therefore, she started omalizumab with a partial reduction of symptoms and exacerbations over time. During a follow-up visit, she showed signs of acute exacerbation and she was switched to benralizumab during her acute phase with a rapid, dramatic amelioration of respiratory symptoms and pulmonary function, without concomitant systemic corticosteroid administration. During the treatment and at follow-up after one month, good tolerance and no side effects were observed.

**Conclusions**: The use of benralizumab seems to be feasible, rapid, and safe in treating acute exacerbation of severe eosinophilic asthma without the use of systemic corticosteroids.

## Abbreviations

ACT, Asthma Control Test; BID, bis in die; FEV1, forced expiratory volume in 1 second, FVC, forced vital capacity; FeNO, fraction of exhaled nitric oxide; LAMA, long-acting muscarinic antagonist; IV, intravenous.

## Introduction

Acute exacerbation of severe asthma is a medical emergency that requires treatment with high-dose corticosteroids and accounts for >60% of the total costs of the disease care, primarily required for emergency visits and hospitalizations ^1, 2^.

Monoclonal antibodies are a relatively new therapeutic option for patients with severe refractory asthma, which can be used as an add-on to the maintenance therapy, with long-term effects such as reducing the need for systemic corticosteroids usage, improving asthma symptoms control and reducing exacerbations ^3^. We report the use of a monoclonal antibody against interleukin-5, benralizumab, for the treatment of an acute exacerbation of severe asthma in a patient who refused to take systemic steroids.

## Case presentation

### Patient information and medical history

We present the case of a 59-year-old non-smoker Caucasian female, lawyer, with a history of allergic asthma since childhood. She had no pets at home.

**Figure 1.**
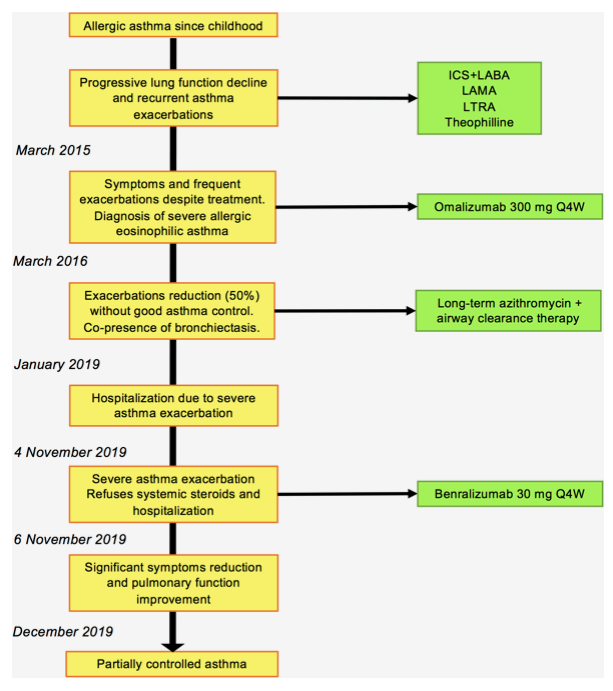
Case report timeline following CARE guidelines. LAMA, long acting muscarinic agonist; LTRA, leukotrienes receptor antagonist; Q4W: every four weeks.

Timeline of patient's clinical evolution with diagnostic tests and treatments is shown in Figure 1.

The patient had a body mass index within the normal range, chronic rhinosinusitis without nasal polyposis, and gastro-esophageal reflux under pharmacological control.

Her basal asthma regimen over the years was budesonide/formoterol 160 mcg/4.5 mcg, four puffs/day, with good treatment adherence and correct inhalation technique.

Over the past years, despite her adequate adherence to her maintenance regimen, the patient experienced a gradual worsening of her asthma symptoms, together with a progressive decline of her lung function and an increasing number of exacerbations, some of which required hospitalization. Therefore, after a pulmonologist consultation, combined treatment with a long-acting muscarinic antagonist (LAMA; 2.5 mcg tiotropium Respimat inhaler, two puffs daily), theophylline (300 mg tablets, bis in die) and a leukotriene receptor antagonist (montelukast, 10 mg daily) were added to her maintenance treatment regimen.

The patient’s adherence to both inhaled (ICS + LABA and LAMA) and oral (theophylline and montelukast) therapy continues to be very good, due to her fear of possible need for intravenous/oral steroids in case of non-adequate adherence.

### Initial presentation, diagnostic tests and treatment

In March 2015, she was referred to our outpatient respiratory clinic at Policlinico Vittorio-Emanuele di Catania, Italy, due to the persistence of asthma symptoms despite the therapy. Pulmonary function tests showed a forced vital capacity (FVC) of 78% of predicted value (2620 mL), and a forced expiratory volume in one second (FEV _1_) of 55% of predicted value (1400 mL), with a post-bronchodilator (after 400 mcg of salbutamol) increase in FEV _1_ of 24% (1990 mL).

She showed sensitization to multiple inhalant allergens by skin prick tests (house dust mites, dog and cat dander, and *Parietaria judaica*), high serum total IgE levels (201 IU/mL), high blood eosinophils count (670 cells/µL) and high fraction of exhaled nitric oxide (FeNO; 51 ppb). She complained of frequent exacerbations of her asthma symptoms and recurrent hospital admissions over the past year (nearly one/month) and, therefore, we classified her as severe refractory asthma according to Global Initiative for Asthma (GINA) guidelines ^4^, after excluding other respiratory diseases that may share common clinical manifestations with severe asthma, as recommended by ERS/ATS guidelines ^5^.

Moreover, the patient had always been reluctant to use systemic corticosteroids, referring to an almost immediate appearance of urinary retention, so she refused the proposed short course of oral corticosteroids. Therefore, we started treatment with omalizumab (300 mg via subcutaneous injections administered every four weeks).

### Subsequent presentations, diagnostic tests and treatments

We further evaluated our patient every four weeks, and she reported a dramatic reduction in the number and severity of exacerbations (nearly 50% less) and the number of hospitalizations overall, without using systemic steroids. Still, she described persistence of respiratory symptoms and poor asthma control with the need of a salbutamol inhaler at least five times a day, despite the maintenance and the biologic therapy. At her follow-up visit after one year from the first dose of omalizumab, her Asthma Control Test (ACT) score was 8, her FeNo was 33 ppb, and her pulmonary function tests showed an FEV _1_ of 73% of predicted value (1860 mL) and FVC of 90% of predicted value (3030 mL), an FEV _1_ / FVC of 61% and a positive post-bronchodilatation test response, with FEV _1_ reaching 85% (+ 12%) and 2105 mL. She underwent a chest computed tomography scan, revealing diffuse bronchiectasis; therefore, she started long-term azithromycin and airway clearance therapy on top of her usual maintenance treatment. The patient still achieved partially controlled asthma with treatment optimization.

In January 2019, she presented to the emergency department for a new severe exacerbation characterized by whistling dyspnea, greenish sputum, and acute respiratory failure, and required hospitalization.

During the hospital stay, her sputum cytological analysis showed 17% of eosinophils out of a total count of 250,000 cells/mL, and her blood tests exhibited an elevated peripheral eosinophilic count (470 cells/µL). Pulmonary function tests revealed an FEV _1_ of 49% of predicted, FVC of 72% of predicted and FEV _1_ / FVC of 57.4%. She started intravenous (IV) theophylline (240 mg intravenous slow bolus followed by a continuous infusion of 0.5 mg/kg/h), IV piperacillin/tazobactam (4.5 g every eight hours), IV corticosteroids (40 mg of methylprednisolone), and oxygen (nasal cannula 2 liters/minute) on top of her maintenance therapy, with progressive symptoms relieved. At discharge, we proposed a switch from omalizumab to mepolizumab, but the patient refused.

On November 4, 2019, she presented for a regular outpatient visit and complained of a worsening of her usual respiratory symptoms, presence of wheezing, reduced tolerance to physical activity, abundant purulent sputum, and frequent nocturnal awakenings. She had taken 25+ puffs of salbutamol to alleviate her chest tightness. Her oxygen saturation was 91% at rest.

Pulmonary function tests showed an FEV _1_ of 61% of predicted value (1480 mL), FVC of 74% of predicted value (2410 mL), and FEV _1_ / FVC of 61%. A post-bronchodilatation test FEV _1_ reached 70% (+9%) and 1700 mL. Her ACT score was 6. She also underwent blood tests, showing elevated eosinophils (390 cells/µL) and her FeNo was 60 ppb.

We proposed a short course of steroids, but she refused to take them due to the fear of urinary retention; she also refused hospitalization. Therefore, as a rescue solution, and due to the presence of elevated eosinophils, we decided to switch omalizumab to benralizumab (30 mg by subcutaneous injection every four weeks for the first three doses, and then every eight weeks thereafter) on top of her maintenance therapy. The same day the patient started the first administration of 30 mg subcutaneous injection of benralizumab.

### Follow-up and outcomes

At a telephone follow-up after 24 hours, she reported a significant improvement in her asthma symptoms with the almost total disappearance of sputum, absence of nocturnal awakening, and noticeable reduction of dyspnea, which made her stop rescue medication. At her 48 hours follow-up visit, the physical examination revealed a dramatic decrease in whistles as compared to the day before. The FEV _1_ was 80% of predicted value (1940 ml, +19% compared to two days before), FVC was 88% of predicted value (2890 ml, +14% compared to two days before) and FEV _1_ / FVC was 67% (+6%). Oxygen saturation has improved too, reaching 98% at rest, and her blood test showed complete depletion of eosinophils in peripheral blood. After four weeks, she returned to our outpatient service for administration of the second dose of benralizumab. She described good control of respiratory symptoms within the last month with no exacerbations, nocturnal awakening, nor sputum, and only occasional use of salbutamol. She did not report any adverse effects. Her blood count continued to show complete eosinophils depletion. Pulmonary function tests showed a further increase in lung function: an FEV _1_ of 98% of predicted value (2360 ml), FVC of 94% of predicted value (3140 ml), and FEV _1_ / FVC 75%. Her ACT reached a score of 18, her highest result ever and her FeNo was 47 ppb. Based on these excellent results the patients was then prescribed with benralizumab 30 mg every four weeks for the following two doses and then every 8 weeks, with the indication of performing a blood test to evaluate the blood eosinophils count before each biologic dose administration.

## Discussion

To the best of our knowledge, this case report is the first in the literature on the use of benralizumab administration during an acute attack of severe refractory eosinophilic asthma, without the concomitant use of systemic steroids. The main finding of this report is the efficacy and safety of benralizumab treatment during the acute phase of eosinophilic asthma exacerbation, showing a terrific and rapid response in terms of improvement of symptoms and pulmonary function (significant gain in FEV _1_ after only 48 hours), and reduction of sputum production, without the concomitant use of systemic corticosteroids and avoiding hospitalization.

Biologics have shown long-term beneficial effects in the management of severe asthma patients ^6^. Characterizing the properties of one molecule versus another might be crucial for a more personalized treatment approach ^7^. The speed of treatment onset might represent an essential underrated characteristic to consider in this context.

Omalizumab has been shown to reduce both asthma symptoms and exacerbations within the first 30 days of treatment ^8^, while mepolizumab in months ^9^ and reslizumab in weeks ^10^. Indeed, benralizumab is responsible for rapid symptomatologic improvement, obtainable after only a few days ^11^.

This brilliant and fast therapeutic effect is due to its antibody-dependent cell-mediated cytotoxicity activity, which results in a complete depletion of eosinophils in both peripheral blood and tissues ^12^.

The rapid improvement in symptoms observed is in line with the results of a post-hoc analysis of two studies (SIROCCO and CALIMA), which showed positive impacts on symptoms by the third day after administration, reducing salbutamol use ^11^. Our report strengthens the data from Nowak and coworkers ^13^, who described similar rapid effects, indicating that the administration of one dose of benralizumab added to usual care in patients who presented with acute asthma to the emergency department decreased asthma exacerbation rate and severity as well as hospitalizations at twelve weeks.

A recent case report ^14^ described a reduction in asthma symptoms after two days and an improvement in respiratory peak flow after four days of benralizumab administration ^14^. Rapid response to treatment was previously described in another report from our group ^15^.

The case presented is the first in which treatment with benralizumab during the acute phase of eosinophilic asthma exacerbation without the concomitant use of systemic steroids, on top of the maintenance treatment regimen, showed rapid resolution of symptoms within 24 hours. The immediate response to the treatment is also supported by a considerable increase in pulmonary function parameters (FEV _1_ +19%) obtained only after 48 hours, even without the concomitant use of systemic steroids. This case could be the starting point for the use of benralizumab in the acute phase of severe eosinophilic asthma exacerbations, starting the treatment early in the emergency room without resorting to systemic steroids. This rapid effect on lung function might be explained by the ability of benralizumab to reduce eosinophils not only in peripheral blood but also in the airways, limiting the release of noxious eosinophil granule proteins in bronchial tissue ^12, 16^.

Moreover, differently from previously published reports ^17, 18^ showing the rapid effects of biologics use together with high doses of systemic steroids and bronchodilators, our report is likely to reveal the real fast effect of benralizumab on acute asthma exacerbation, being used alone on top of the maintenance therapy, without concomitant use of systemic steroids.

This case report has some limitations. We did not report a long-term follow-up. Therefore, we cannot document long-term beneficial effects and treatment tolerance.

In conclusion, benralizumab may represent a feasible treatment as an add-on therapy in the management of acute asthma attacks, even without the use of systemic corticosteroids. Adequately powered multicenter trials are needed to confirm our observation.

## Data availability

All data underlying the results are available as part of the article and no additional source data are required.

## Consent

Written informed consent for publication of clinical details was obtained by the patient.
