# Supplementary material for: Case Report: Acute effect of benralizumab on asthma exacerbation without concomitant corticosteroid use
Source: F1000Res. 2020 Jul 23;9:637. Originally published 2020 Jun 23. [Version 2] doi: 10.12688/f1000research.24603.2 (PMC7443779; doi:10.12688/f1000research.24603.2)
Supplement: Peer review history

# Peer review: Reviewer response for version 2

**DOI:** 10.5256/f1000research.28193.r67879

**Published:** 2020-08-20

**Authors:** Paula Kauppi

**Affiliations:** 1 Inflammation Centre, Skin and Allergy Hospital, Helsinki University Hospital, University of Helsinki, Helsinki, Finland

I have no further comments.

Are enough details provided of any physical examination and diagnostic tests, treatment given and outcomes?

Partly

Is the case presented with sufficient detail to be useful for other practitioners?

Partly

Is sufficient discussion included of the importance of the findings and their relevance to future understanding of disease processes, diagnosis or treatment?

Partly

Is the background of the case’s history and progression described in sufficient detail?

Partly

Reviewer Expertise:

NA

I confirm that I have read this submission and believe that I have an appropriate level of expertise to confirm that it is of an acceptable scientific standard.

# Peer review: Reviewer response for version 1

**DOI:** 10.5256/f1000research.27141.r65420

**Published:** 2020-07-08

**Authors:** Claudio Micheletto

**Affiliations:** 1 Cardio-Thoracic Dept, Respiratory Unit, Integrated University Hospital, Verona, Italy

The Authors presented a case report on the use of benralizumab administration during an acute attack of severe refractory eosinophilic asthma, without the concomitant use of systemic steroids. Benralizumab is an anti-receptor monoclonal anti-IL-5 antibody, which has been shown to be particularly effective in reducing exacerbations, used as regular therapy in severe eosinophilic asthma.

 Some questions:

- How was the patient's adherence to inhalation therapy?
- Did the patient have a previous accurate diagnosis of severe asthma?
- Was the patient then regularly treated with benralizumab?
- Were eosinophils measured in the following weeks?

Are enough details provided of any physical examination and diagnostic tests, treatment given and outcomes?

Yes

Is the case presented with sufficient detail to be useful for other practitioners?

Yes

Is sufficient discussion included of the importance of the findings and their relevance to future understanding of disease processes, diagnosis or treatment?

Yes

Is the background of the case’s history and progression described in sufficient detail?

Yes

Reviewer Expertise:

severe asthma

I confirm that I have read this submission and believe that I have an appropriate level of expertise to confirm that it is of an acceptable scientific standard.

## References

[1] : A randomized trial of benralizumab, an antiinterleukin 5 receptor α monoclonal antibody, after acute asthma. *Am J Emerg Med*.2015;33(1) : 10.1016/j.ajem.2014.09.036 14-20 10.1016/j.ajem.2014.09.036 25445859

# Response

**Published:** 2020-07-17

**Authors:** Claudia Crimi

**Affiliations:** University Hospital Policlinico - Vittorio Emanuele, Italy

Dear Dr. Micheletto,

 Thank you for your time and consideration in reviewing our article.

 We are glad that you find our case report interesting.  We were thrilled to read the approval from an expert in the field like you.

 We have now added a bit more information as you requested. 

 Best Regards,

 Claudia Crimi, MD, PhD 

 ___________________________

**Specific point-by-point response:**

 The Authors presented a case report on the use of benralizumab administration during an acute attack of severe refractory eosinophilic asthma, without the concomitant use of systemic steroids. Benralizumab is an anti-receptor monoclonal anti-IL-5 antibody, which has been shown to be particularly effective in reducing exacerbations, used as regular therapy in severe eosinophilic asthma.

 ·        How was the patient's adherence to inhalation therapy?

**Reply:**Thank you for your comment. Based on your suggestion we have added a sentence stating that patient's adherence to both inhaled (ICS + LABA and LAMA) and oral (theophylline and Montelukast) therapy, continue to be very good, also due to her fear of possible need for intravenous/oral steroids in case of non-adequate adherence.

 ·        Did the patient have a previous accurate diagnosis of severe asthma?

**Reply:**Thank you for your comment. We diagnosed severe asthma according to ERS/ATS guidelines [1]; therefore, we excluded other respiratory diseases that may share common clinical manifestations of severe asthma (i.e., bronchopulmonary aspergillosis, vasculitis, chronic cough). We have now specified it in the text and added the appropriate reference as below.

  

1. Chung KF, Wenzel SE, Brozek JL, et al.: International ERS/ATS guidelines on definition, evaluation and treatment of severe asthma. Eur Respir J. 2014; 43(2): 343–373.

 

 ·        Was the patient then regularly treated with benralizumab?

**Reply: **Thank you for pointing out this aspect. Yes, the patient was then treated regularly with benralizumab 30 mg subcutaneously every four weeks for the first three doses and then every eight weeks. We have better clarified this in the text (paragraph follow-up and outcomes, last sentence).

 ·        Were eosinophils measured in the following weeks?

**Reply:**Thank you for highlighting this aspect. Yes, we executed blood eosinophils count 48h and exactly four weeks after the first benralizumab administration (right before the second dose), showing a complete eosinophils depletion in both determinations (as described in the paragraph follow-up and outcomes). Moreover, a blood test was performed before every next benralizumab dose. We have now better specify this aspect in the text (paragraph follow-up and outcomes, last sentence).

# Peer review: Reviewer response for version 1

**DOI:** 10.5256/f1000research.27141.r65647

**Published:** 2020-07-06

**Authors:** Paula Kauppi

**Affiliations:** 1 Inflammation Centre, Skin and Allergy Hospital, Helsinki University Hospital, University of Helsinki, Helsinki, Finland

This is a case report of a patient who received a dose of benralizumab for acute asthma. The patient had been on omalizumab as an add on therapy with ICS, LABA, LAMA, montelukast and theophylline. She refused per oral glucocorticoids. She had moderate to severe obstruction in spirometry and bronchodilator response. She received a dose of benralizumab when having an asthma exacerbation. Two days later her asthma symptoms had decreased and lung function increased.

1. What was adherence of the patient with her asthma medication (ICS, LABA, LAMA, montelukast and theophylline)?
2. Did the patient smoke regularly or had she ever smoked regularly?
3. Did the patient have pets at home?
4. How many exacerbations or severe exacerbations the patient had had a year before she started omalizumab?
5. How many exacerbations or severe exacerbations the patient had had a year before she started benralizumab?
6. In previous studies, benralizumab has been shown to reduce number of eosinophils in 24 hours and to increase FEV1 in 4 weeks. In this case report, benralizumab seemed to increase FEV1 in two days? Could you discuss the unexpected rapid changes?
7. Did the patient receive some other asthma drugs or were the other asthma drugs changed on the same visit when benralizumab was started?
8. Please consider these two reports on use of biologicals in acute asthma: Renner A, Marth K, Schäffl-Doweik L, Pohl W. Case Report: Reslizumab in an Invasively Ventilated Patient With Acute Respiratory Failure. J Allergy Clin Immunol Pract. 2019;7(8):2922-2923. doi:10.1016/j.jaip.2019.05.019 ^1^ and Milger K, Schroeder I, Behr J, Meis T, Wulffen WV, Kneidinger N. Omalizumab Rescue Therapy for Refractory Status Asthmaticus. Ann Intern Med. 2019;170(5):351-352. doi:10.7326/L18-0359 ^2^.

Are enough details provided of any physical examination and diagnostic tests, treatment given and outcomes?

Partly

Is the case presented with sufficient detail to be useful for other practitioners?

Partly

Is sufficient discussion included of the importance of the findings and their relevance to future understanding of disease processes, diagnosis or treatment?

Partly

Is the background of the case’s history and progression described in sufficient detail?

Partly

Reviewer Expertise:

Asthma, obstructive pulmonary disease, bronchiectasis, allergic diseases

I confirm that I have read this submission and believe that I have an appropriate level of expertise to confirm that it is of an acceptable scientific standard, however I have significant reservations, as outlined above.

## References

[1] : Reslizumab in an invasively ventilated patient with acute respiratory failure. *The Journal of Allergy and Clinical Immunology: In Practice*.2019;7(8) : 10.1016/j.jaip.2019.05.019 2922-2923 10.1016/j.jaip.2019.05.019 31136820

[2] : Omalizumab Rescue Therapy for Refractory Status Asthmaticus. *Annals of Internal Medicine*.2019;170(5) : 10.7326/L18-0359 10.7326/L18-0359 30458534

# Response

**Published:** 2020-07-18

**Authors:** Claudia Crimi

**Affiliations:** University Hospital Policlinico - Vittorio Emanuele, Italy

Dear Dr. Kauppi,

 Thank you for your time and consideration in reviewing our article. 

 Your comments were very helpful to improve the manuscript and we hope that the revised version makes it now acceptable for you. 

 We hereby provide a point-by-point reply to your comments.

 We look forward hearing from you soon.

 Best Regards,

 Claudia Crimi, MD, PhD 

 ______________________

 This is a case report of a patient who received a dose of benralizumab for acute asthma. The patient had been on omalizumab as an add on therapy with ICS, LABA, LAMA, montelukast and theophylline. She refused per oral glucocorticoids. She had moderate to severe obstruction in spirometry and bronchodilator response. She received a dose of benralizumab when having an asthma exacerbation. Two days later her asthma symptoms had decreased and lung function increased.

 1.     What was adherence of the patient with her asthma medication (ICS, LABA, LAMA, montelukast and theophylline)?

**Reply:**Thank you for your comment. Based on your suggestion we have added a sentence stating that the patient’s adherence to both inhaled (ICS + LABA and LAMA) and oral (theophylline and montelukast) therapy continues to be very good, due to her fear of possible need for intravenous/oral steroids in case of non-adequate adherence (see the section “Patient information and medical history” last sentence).

 2.     Did the patient smoke regularly or had she ever smoked regularly?

**Reply:**Thanks for pointing out this. No, the patient has never smoked; this information is present in the first sentence of the paragraph “Patient information and medical history”.

 3.     Did the patient have pets at home?

**Reply:**Thanks for this comment. No, the patient has never had pets at home; we have now clarified this in the description of patient’s medical history (Second sentence of the paragraph “Patient information and medical history”).

 4.     How many exacerbations or severe exacerbations the patient had had a year before she started omalizumab?

**Reply:**Thanks for highlighting this aspect. As stated in the section “Initial presentation, diagnostic tests and treatment” (please see second paragraph) the patient complained one exacerbation per month (12 exacerbations/year) over the past year before staring omalizumab treatment.

 5.     How many exacerbations or severe exacerbations the patient had had a year before she started benralizumab? 

**Reply:**Thanks for highlighting this aspect. As stated in the section “Subsequent presentation, diagnostic tests and treatments” (please see first sentence) the patient reported a reduction of nearly 50% of exacerbation after one year of omalizumab (one exacerbation every 2 months - 6 exacerbations/year) before staring benralizumab treatment.

 6.   In previous studies, benralizumab has been shown to reduce number of eosinophils in 24 hours and to increase FEV1 in 4 weeks. In this case report, benralizumab seemed to increase FEV1 in two days? Could you discuss the unexpected rapid changes?

**Reply:**The patient showed a complete depletion in peripheral blood eosinophil count within 2 days after switching from omalizumab to benralizumab, showing an increase of pre-bronchodilator FEV1% (+19%), FVC (+14%) and FEV1/FVC (6%). 

 This rapid eosinophils reduction is consistent with previous benralizumab studies [1] and reflects its mechanism. Moreover, benralizumab has shown to significantly reduce eosinophils not only in peripheral blood but also in airways, limiting the release of noxious eosinophil granule proteins in bronchial tissues [2,3]. IL-5 is upstream of IgE in the canonical allergic inflammatory cascade, coupled with current emerging evidence of non-IgE-mediated alternative IL-5 pathways that can maintain eosinophilia is reasonable to expect an anti-IL-5R mAb to be effective when an anti-IgE mAb fails to curb symptoms [3].  We suggest that these mechanisms have been responsible for the rapid improvement of the patient's lung function. In our patient, eosinophils reduction allowed the tapering of type-2 inflammatory processes accountable for her asthma exacerbations. These considerations are corroborated by the good control of respiratory symptoms, and further respiratory function improvements 4 weeks after the first benralizumab administration.  Therefore, our observation confirms that the therapeutic responses to anti-asthma biological drugs are strictly individual, being dependent on each patient's specific endotype.  We have discussed this point in the discussion section, as requested.

  

1. Nowak RM, Parker JM, Silverman RA, et al. A randomized trial of benralizumab, an anti interleukin 5 receptor αmonoclonal antibody, after acute asthma. *Am J Emerg Med*. 2015;33(1):14-20. doi:10.1016/j.ajem.2014.09.036 (REF 13)
2. Laviolette M, Gossage DL, Gauvreau G, et al.: Effects of benralizumab on airway eosinophils in asthmatic patients with sputum eosinophilia. J Allergy Clin Immunol. 2013; 132(5): 1086–1096 e1085 (REF 12)
3. Mukherjee M, Bakakos P, Loukides S. New paradigm in asthma management: Switching between biologics!. *Allergy*. 2020;75(4):743-745. doi:10.1111/all.14038 (REF 16)

 

 7.     Did the patient receive some other asthma drugs or were the other asthma drugs changed on the same visit when benralizumab was started?

**Reply:**Thank you for highlighting this aspect. No. The patient at this point was already taking high-dose ICS+LABA, LAMA, montelukast, theophylline, omalizumab (last administration exactly 4 weeks prior), azithromycin and 25+ daily puffs of salbutamol. During the visit, due to presence of wheezing, reduced tolerance to physical activity, abundant purulent sputum, frequent nocturnal awakenings and rest oxygen saturation of 91%, her reluctance to be hospitalized and to take oral or systemic corticosteroids, and because of the presence of 390 eosinophils/µL we decided to immediately switch from omalizumab to benralizumab 30mg. We have now clarified this aspect adding a sentence at the end of the section “Subsequent presentations, diagnostic tests and treatments” (second-last sentence).

 8. Please consider these two reports on use of biologicals in acute asthma: Renner A, Marth K, Schäffl-Doweik L, Pohl W. Case Report: Reslizumab in an Invasively Ventilated Patient With Acute Respiratory Failure. J Allergy Clin Immunol Pract. 2019;7(8):2922-2923. doi:10.1016/j.jaip.2019.05.019 ^1^and Milger K, Schroeder I, Behr J, Meis T, Wulffen WV, Kneidinger N. Omalizumab Rescue Therapy for Refractory Status Asthmaticus. Ann Intern Med. 2019;170(5):351-352. doi:10.7326/L18-0359 ^2^.

**Reply:**Thank you for referring to these interesting references. Both reports showed the rapid effects of biologics, but, as stated by the authors, it is not possible to exclude that patients would have improved anyway due to the high doses of systemic bronchodilators and corticosteroids not possible to establish the effect of biologic alone. Our case is the first report of an acute asthma exacerbation treated with benralizumab without the addition of systemic corticosteroid, antibiotic or aggressive bronchodilator therapy, being benralizumab administered on top of patient’s maintenance treatment. Therefore, in our report, it is likely to verify and measure the real effect of benralizumab alone during an acute asthma exacerbation, with complete eosinophils reduction and pulmonary function improvement only after 48 hours benralizumab 30 mg subcutaneous administration. We have now discussed the references as suggested.
